# Supplementary material for: Characterization of Lysine Methylation During Neuronal Differentiation of LUHMES cells
Source: bioRxiv. 2026 Jan 2:2025.12.31.696910. Preprint. [Version 1] doi: 10.64898/2025.12.31.696910 (PMC12776253; doi:10.64898/2025.12.31.696910)
Supplement: 1 [file NIHPP2025.12.31.696910V1-supplement-1.pdf]

bioRxiv preprint doi: https://doi.org/10.64898/2025.12.31.696910; this version posted January 2, 2026. The copyright holder for this preprint (which was not certified by peer review) is the author/funder, who has granted bioRxiv a license to display the preprint in perpetuity. It is made available under aCC-BY 4.0 International license.

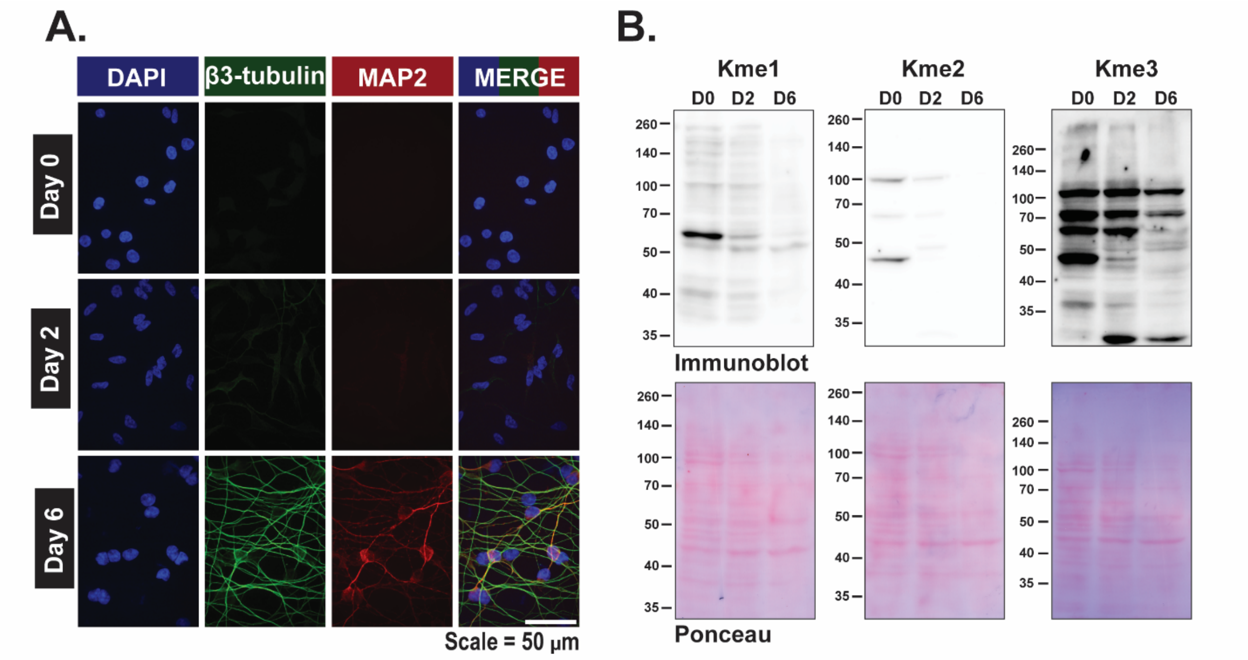

Figure S1: Characterization of LUHMES cell differentiation. (A) Immunocytochemistry of undifferentiated, day 2 differentiated, and day 6 differentiated LUHMES cells: β3-tubulin (green), MAP2 (red), and Nucleus (Blue). Scale bar represents 50 µm. **(B)** *In vitro* changes in lysine methylation of non-histone proteins across LUHMES differentiation days 0, 2, and 6 (D0, D2, D6) detected via immunoblotting using pan-lysine methyl antibodies against all three methyl states, mono- (Kme1), di- (Kme2), and tri- (Kme3) methylation. Top panel depicts immunoblotting (25 µg LUHMES lysate loaded per lane) and bottom panel depicts Ponceau Red staining.

bioRxiv preprint doi: https://doi.org/10.64898/2025.12.31.696910; this version posted January 2, 2026. The copyright holder for this preprint (which was not certified by peer review) is the author/funder, who has granted bioRxiv a license to display the preprint in perpetuity. It is made available under aCC-BY 4.0 International license.

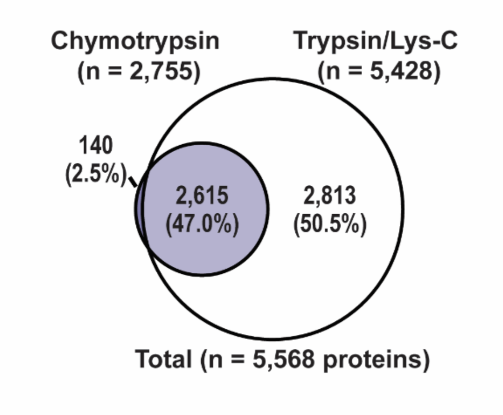

Figure S2: Comparison of the LUHMES proteome from the trypsin/lys-C and chymotrypsin experiments. Venn diagram depicting overlap of proteins quantified in the trypsin/lys-C and chymotrypsin experiments.

bioRxiv preprint doi: https://doi.org/10.64898/2025.12.31.696910; this version posted January 2, 2026. The copyright holder for this preprint (which was not certified by peer review) is the author/funder, who has granted bioRxiv a license to display the preprint in perpetuity. It is made available under aCC-BY 4.0 International license.

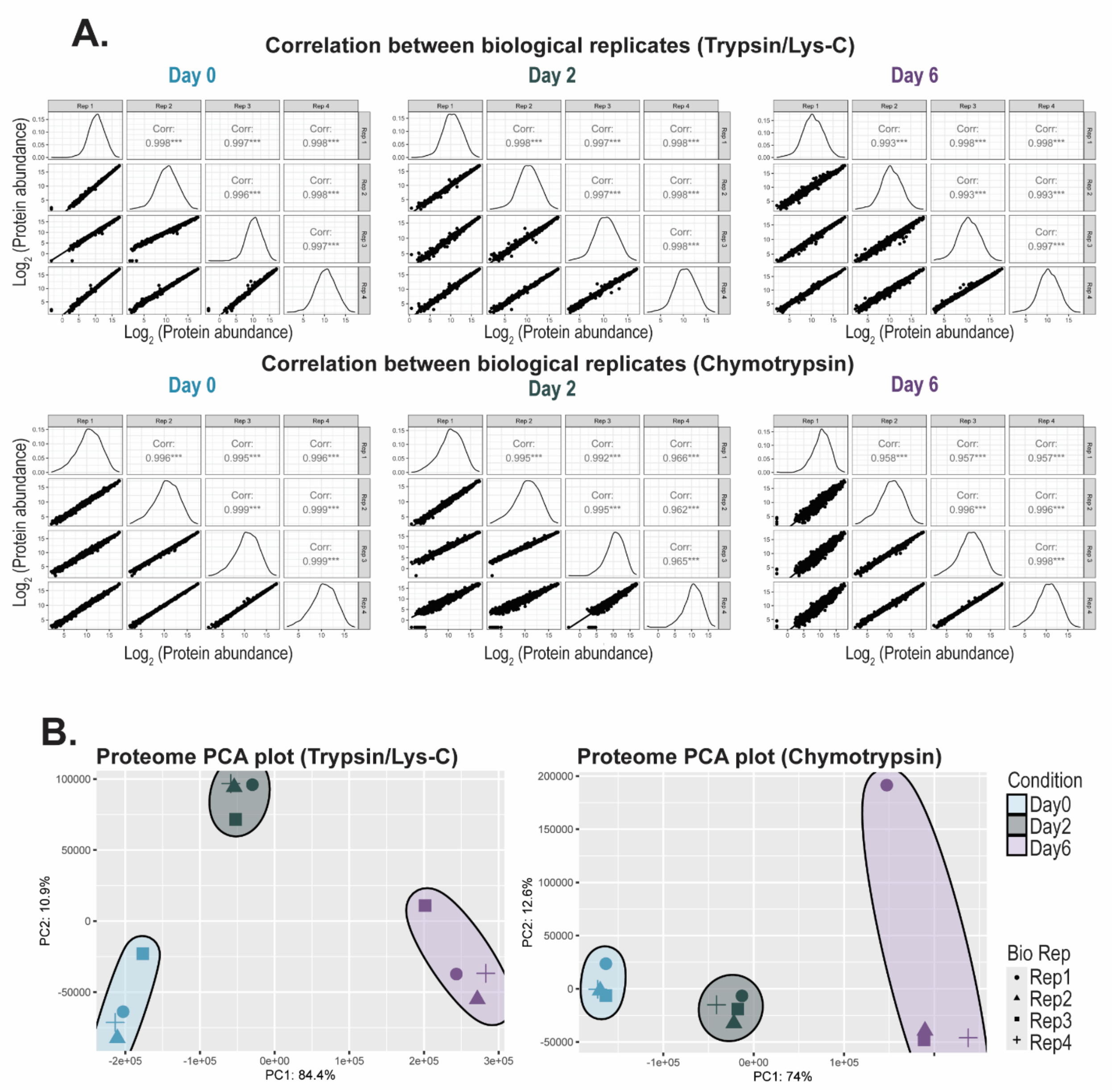

Figure S3: Correlation of protein abundance across biological replicates within the trypsin/lys-C and chymotrypsin experiments. (A) Pearson correlation analysis of the average $\log_2$ abundance of quantified proteins between biological replicates within the trypsin/lys-C experiment (n=5,428) and within the chymotrypsin experiment (n=2,755). **(B)** Principal component analyses (PCA) of the trypsin/lys-C and chymotrypsin proteomes. Each data point shape represents a different biological replicate.

bioRxiv preprint doi: https://doi.org/10.64898/2025.12.31.696910; this version posted January 2, 2026. The copyright holder for this preprint (which was not certified by peer review) is the author/funder, who has granted bioRxiv a license to display the preprint in perpetuity. It is made available under aCC-BY 4.0 International license.

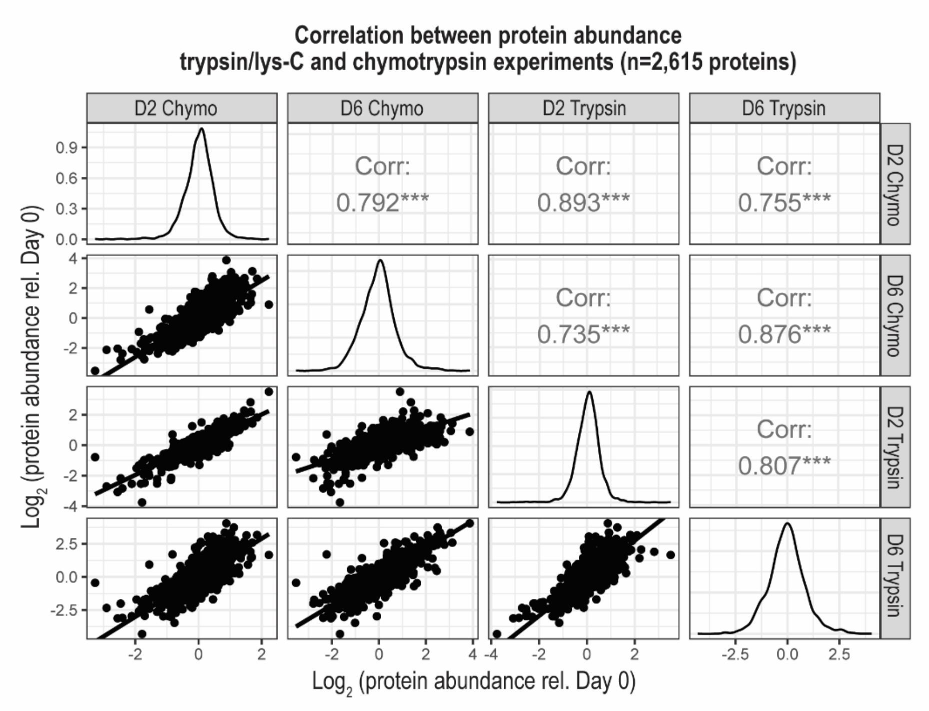

Figure S4: Correlation between abundance of proteins quantified in the trypsin/lys-C and chymotrypsin experiments. Pearson correlation analysis of the average $\log_2$ protein abundance on differentiation days 2 and 6 relative to day 0 for proteins quantified in both the trypsin/lys-C and chymotrypsin experiments (n=2,615).

bioRxiv preprint doi: https://doi.org/10.64898/2025.12.31.696910; this version posted January 2, 2026. The copyright holder for this preprint (which was not certified by peer review) is the author/funder, who has granted bioRxiv a license to display the preprint in perpetuity. It is made available under aCC-BY 4.0 International license.

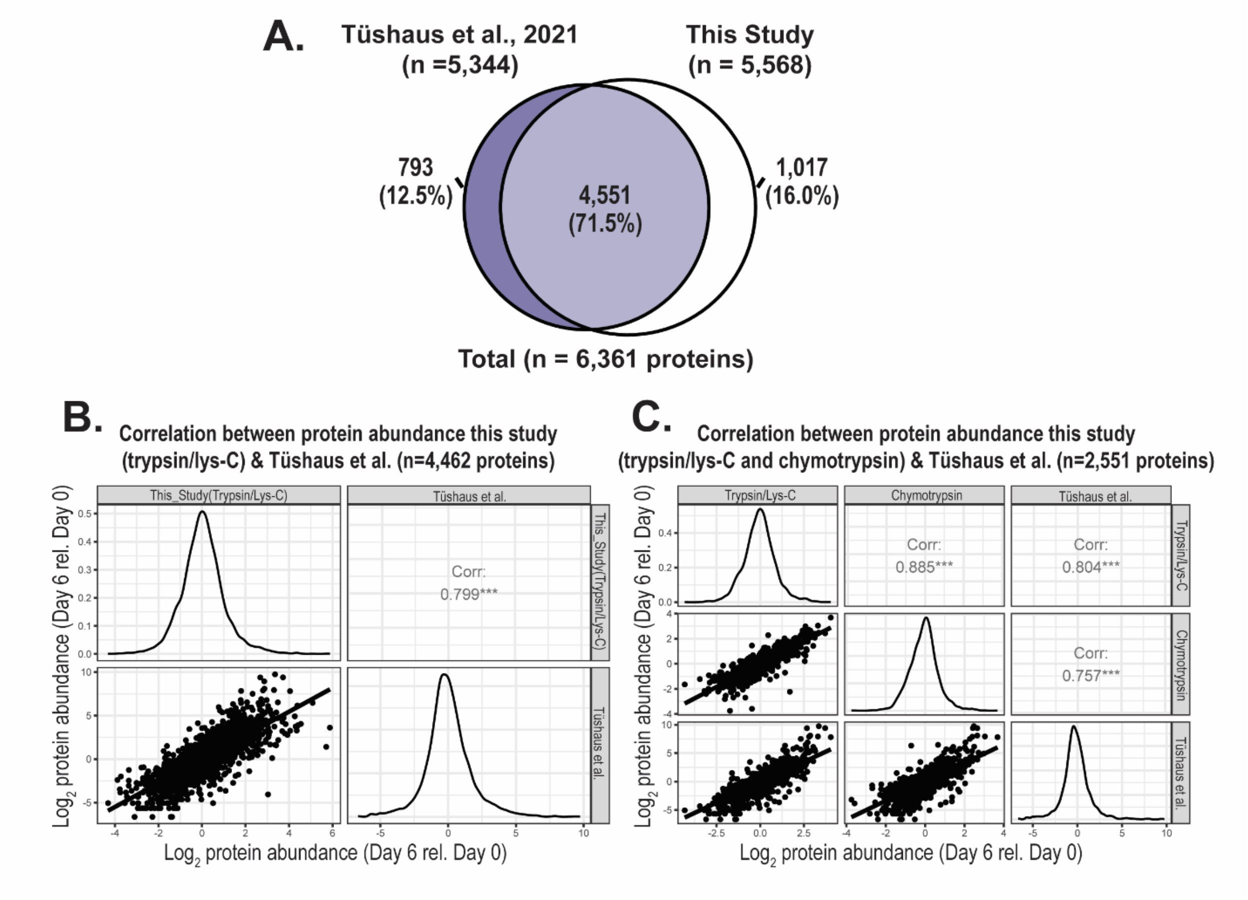

Figure S5: Correlation between the LUHMES proteome from this study and Tüshaus et al. (A) Venn diagram depicting overlap of proteins quantified in this study and Tüshaus et al. **(B)** Pearson correlation analysis of the average $\log_2$ protein abundance on differentiation day 6 relative to day 0 of proteins quantified in Tüshaus et al. and our trypsin/lys-C experiment (n=4,462), and **(C)** proteins quantified in Tüshaus et al. and our trypsin/lys-C and chymotrypsin experiments (n=2,551).

bioRxiv preprint doi: https://doi.org/10.64898/2025.12.31.696910; this version posted January 2, 2026. The copyright holder for this preprint (which was not certified by peer review) is the author/funder, who has granted bioRxiv a license to display the preprint in perpetuity. It is made available under aCC-BY 4.0 International license.

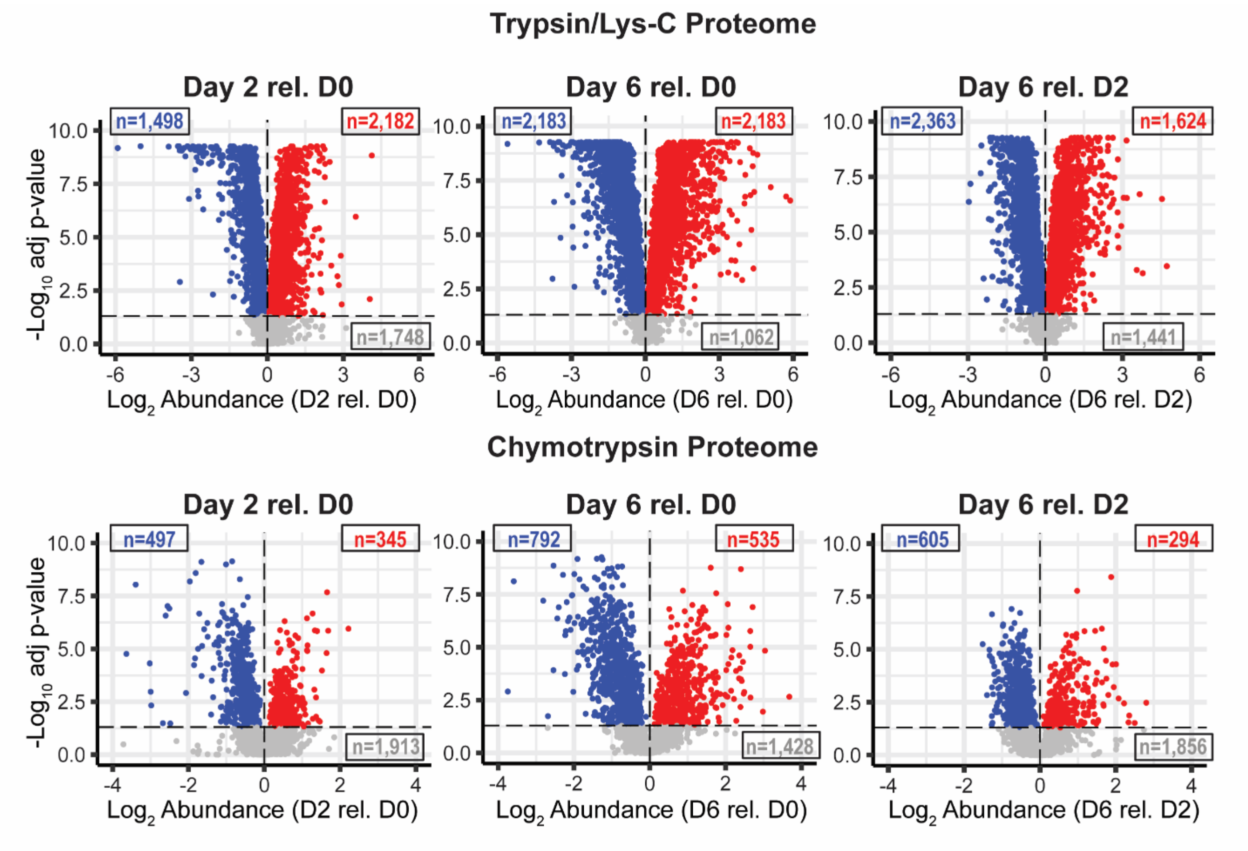

Figure S6: Global protein abundance changes across LUHMES differentiation. Volcano plots displaying log_2 protein abundance (x-axis) at one day of differentiation relative to another, as depicted. P-values plotted on the y-axis. Significantly (ANOVA padj<0.05 followed by Tukey pairwise padj<0.05) upregulated and downregulated proteins are colored in *red* and *blue*, respectively, while proteins with no statistical change in abundance are colored in *grey*.

bioRxiv preprint doi: https://doi.org/10.64898/2025.12.31.696910; this version posted January 2, 2026. The copyright holder for this preprint (which was not certified by peer review) is the author/funder, who has granted bioRxiv a license to display the preprint in perpetuity. It is made available under aCC-BY 4.0 International license.

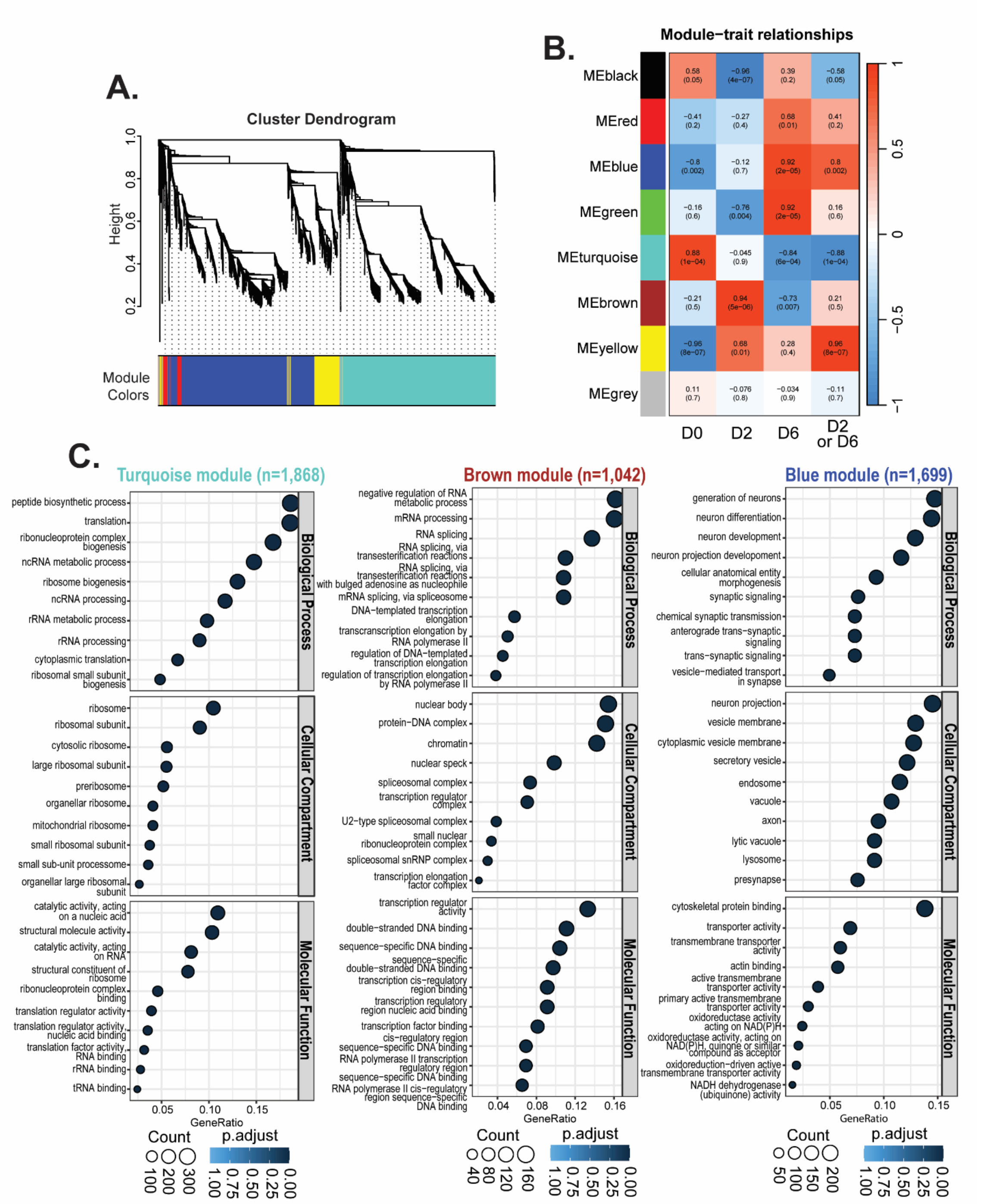

Figure S7: Distinct protein co-expression modules and associated functions revealed through WGCNA and GO terms analyses. (A) WGCNA dendrogram and **(B)** module-trait relationship correlation of protein co-expression modules identified by WGCNA analysis of all proteins quantified in the trypsin/lys-C experiment. **(C)** Enriched GO terms for proteins associated with the following modules: turquoise (enriched expression differentiation day 0), brown (enriched expression differentiation day 2), and blue (enriched expression differentiation day 6).

bioRxiv preprint doi: https://doi.org/10.64898/2025.12.31.696910; this version posted January 2, 2026. The copyright holder for this preprint (which was not certified by peer review) is the author/funder, who has granted bioRxiv a license to display the preprint in perpetuity. It is made available under aCC-BY 4.0 International license.

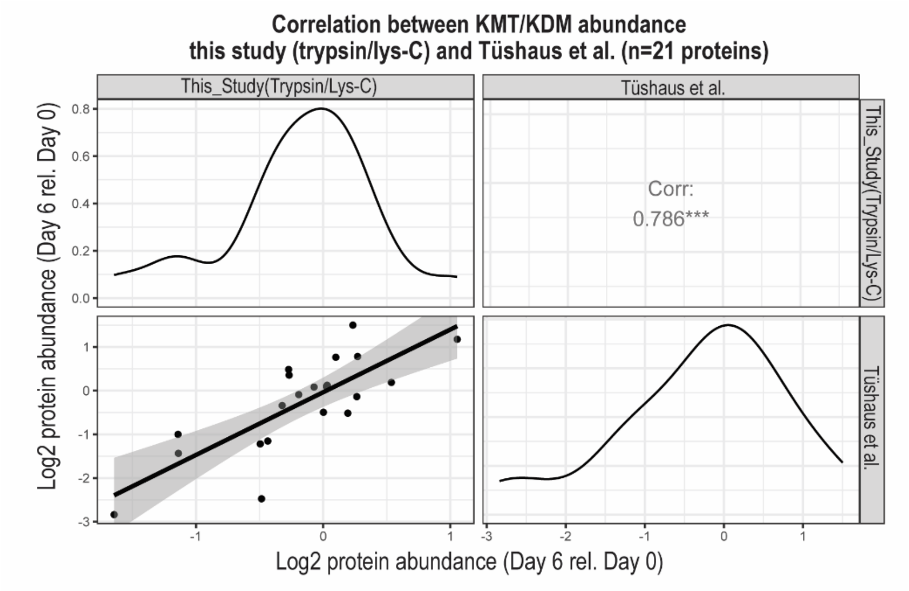

Figure S8: Correlation between abundance of KMTs/KDMs quantified in this study and Tüshaus et al. Pearson correlation analysis of the average $\log_2$ abundance of KMTs/KDMs quantified in both the trypsin/lys-C experiment within this study and in Tüshaus et al. on differentiation day 6 relative to day 0 (n=21).

bioRxiv preprint doi: https://doi.org/10.64898/2025.12.31.696910; this version posted January 2, 2026. The copyright holder for this preprint (which was not certified by peer review) is the author/funder, who has granted bioRxiv a license to display the preprint in perpetuity. It is made available under aCC-BY 4.0 International license.

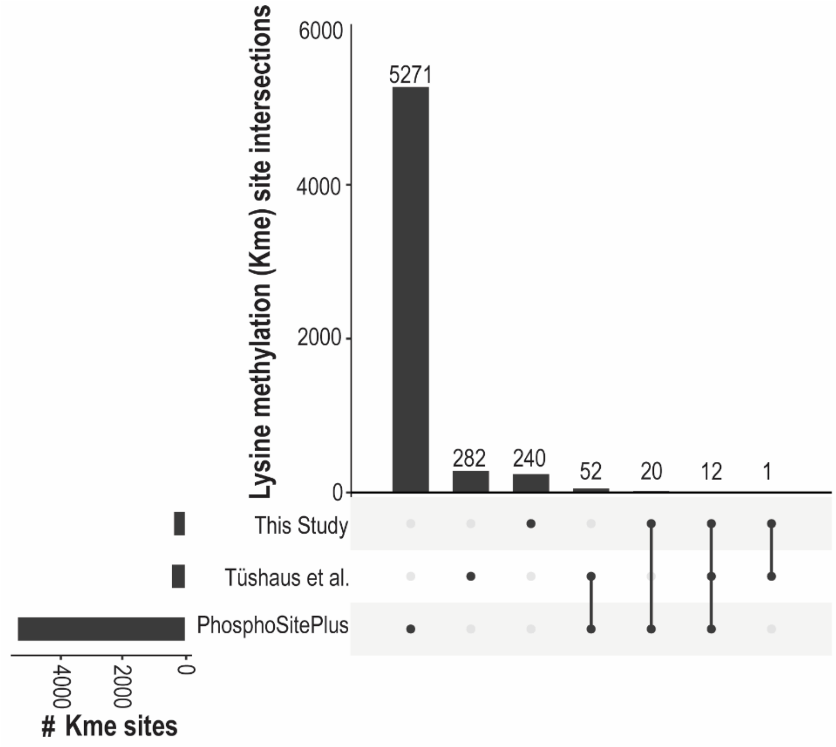

Figure S9: Comparison of Kme sites detected in this study and those reported in the literature. Upset plot displaying lysine methylation sites detected in this study, those reported in the PhosphoSitePlus repository, and those identified in Tüshaus et al. Horizontal bars on the left display the total number of lysine methylation sites identified. Vertical lines connecting points represent overlap of identified Kme sites.

bioRxiv preprint doi: https://doi.org/10.64898/2025.12.31.696910; this version posted January 2, 2026. The copyright holder for this preprint (which was not certified by peer review) is the author/funder, who has granted bioRxiv a license to display the preprint in perpetuity. It is made available under aCC-BY 4.0 International license.

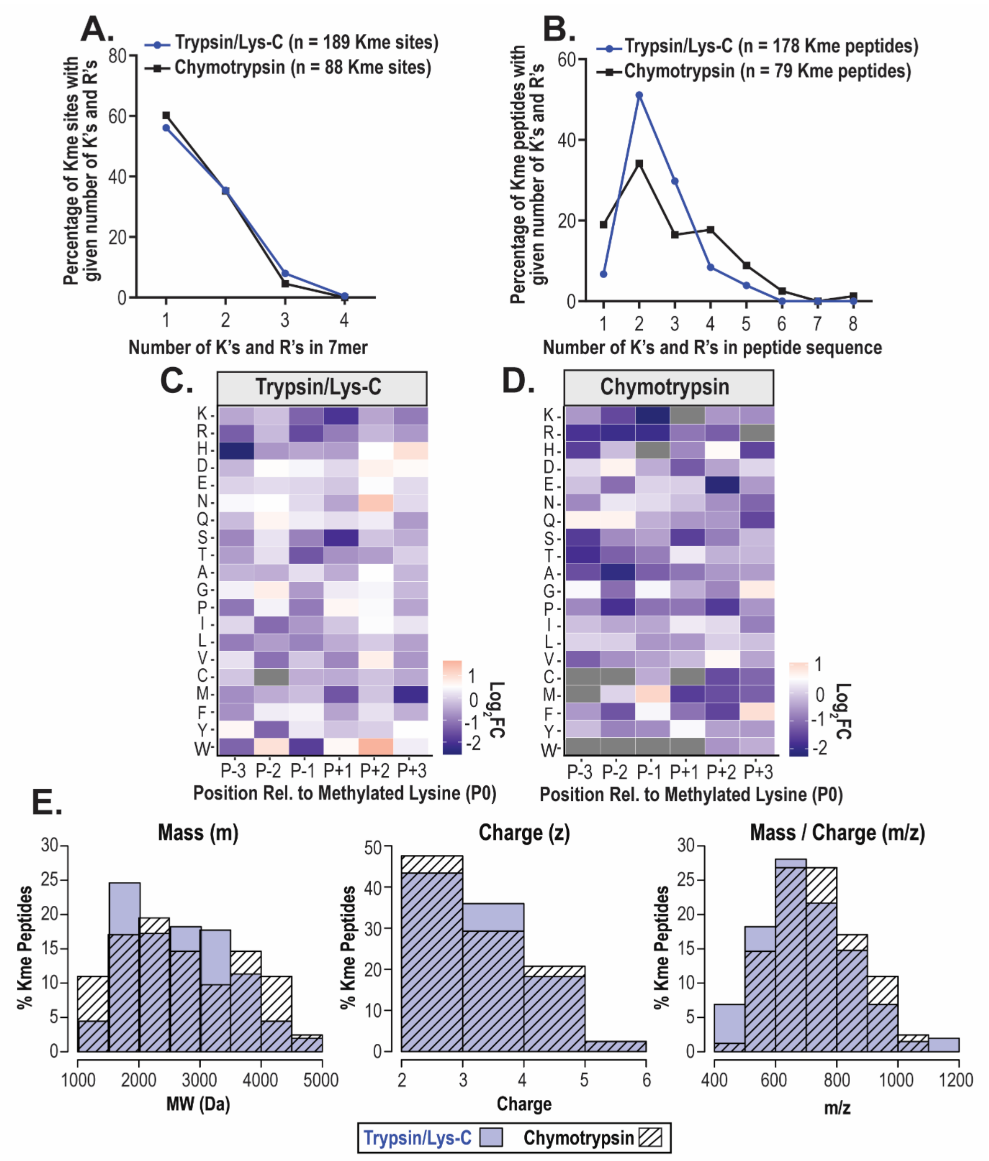

Figure S10: Properties of Kme sites and peptides detected in the trypsin/lys-C and chymotrypsin experiments. Lysine (K) and arginine (R) density of **(A)** 7-mer sequences surrounding methylated lysine residues and **(B)** Kme peptide sequences detected in the mass spectrometry experiments following digestion with trypsin/lys-C (blue) or chymotrypsin (black). **(C)** Trypsin/lys-C and **(D)** chymotrypsin heatmaps depicting the $\log_2$ ratio of the frequency of amino acids within 7-mer motifs surrounding methylated lysine residues compared to the frequency of amino acids within all lysine-centered 7-mer motifs within the human proteome. Gray squares indicate that the amino acid in that position was not present in 7-mer motifs from our study. **(E)** Histograms depicting percentages of Kme peptides detected by mass spectrometry with a given mass (m), charge (z), or mass-to-charge ratio (m/z) following digestion with trypsin/lys-C (light purple bars) or chymotrypsin (white bars with black horizontal stripes).

bioRxiv preprint doi: https://doi.org/10.64898/2025.12.31.696910; this version posted January 2, 2026. The copyright holder for this preprint (which was not certified by peer review) is the author/funder, who has granted bioRxiv a license to display the preprint in perpetuity. It is made available under aCC-BY 4.0 International license.

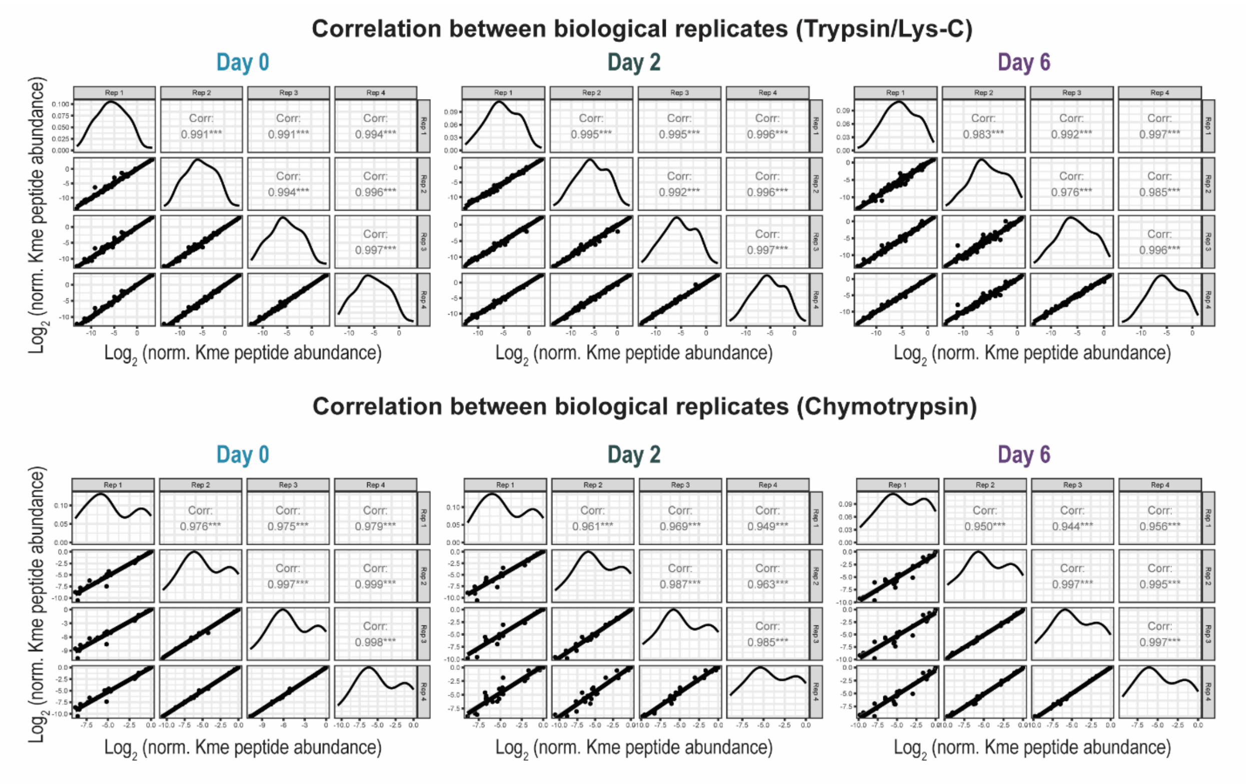

Figure S11: Correlation of Kme peptide abundance across biological replicates within the trypsin/lys-C and chymotrypsin experiments. Pearson correlation analysis of the average $\log_2$ normalized abundance of quantified Kme peptides between biological replicates within the trypsin/lys-C experiment (n=120) and within the chymotrypsin experiment (n=26).

bioRxiv preprint doi: https://doi.org/10.64898/2025.12.31.696910; this version posted January 2, 2026. The copyright holder for this preprint (which was not certified by peer review) is the author/funder, who has granted bioRxiv a license to display the preprint in perpetuity. It is made available under aCC-BY 4.0 International license.

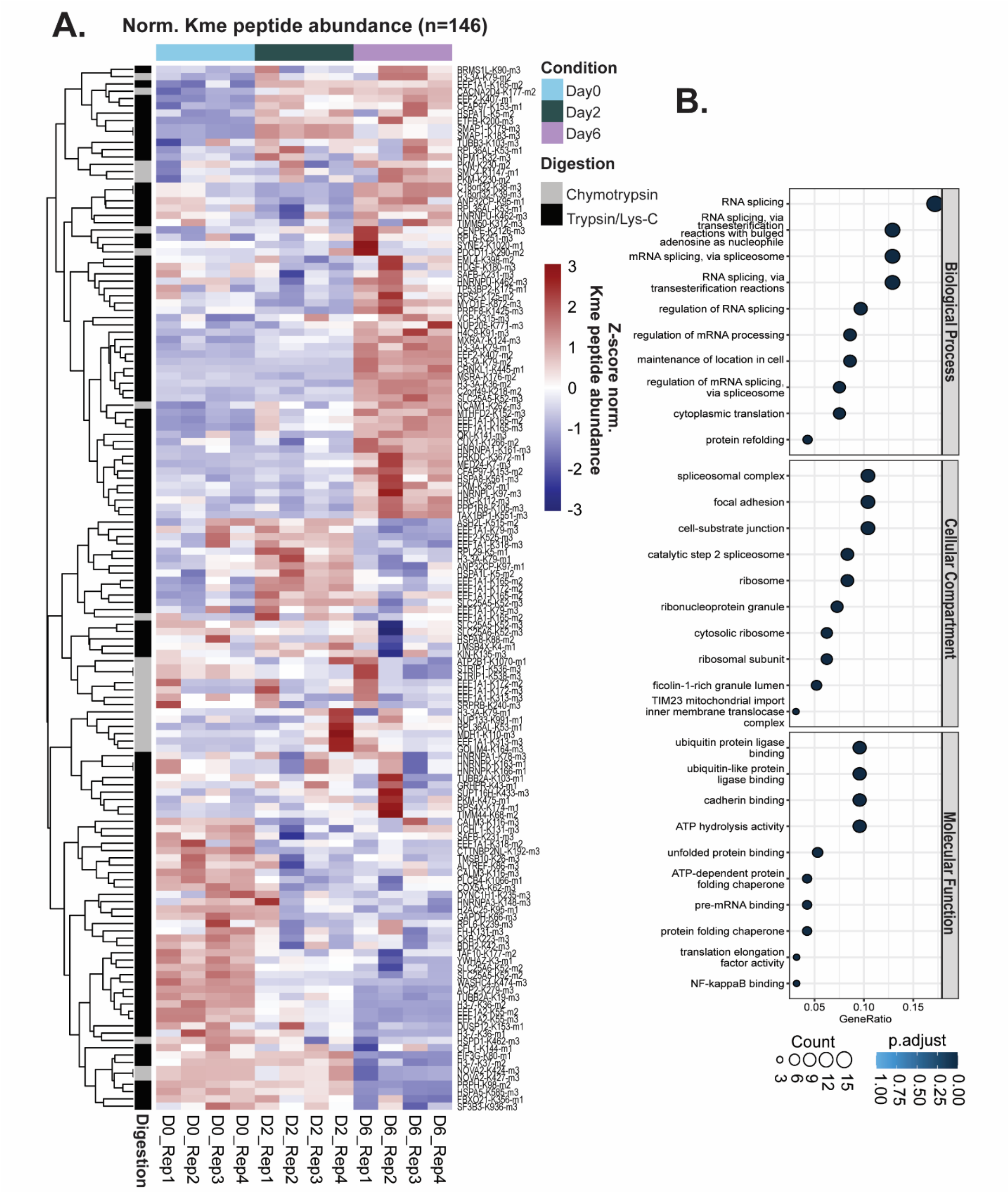

Figure S12: Heatmap of all Kme sites quantified in this study. (A) Heatmap of all Kme peptides (n=146) corresponding to 127 unique Kme sites quantified across LUHMES differentiation. Colors represent the z-score of normalized Kme peptide abundance. Rows (Kme sites) and columns (differentiation sample) are clustered by Euclidian distance. Represented to the left of the rows is the digestion strategy following which the Kme peptide was quantified. **(B)** Enriched gene ontology (GO) terms for 98 unique proteins corresponding to the Kme sites ($p<0.05$).

bioRxiv preprint doi: https://doi.org/10.64898/2025.12.31.696910; this version posted January 2, 2026. The copyright holder for this preprint (which was not certified by peer review) is the author/funder, who has granted bioRxiv a license to display the preprint in perpetuity. It is made available under aCC-BY 4.0 International license.

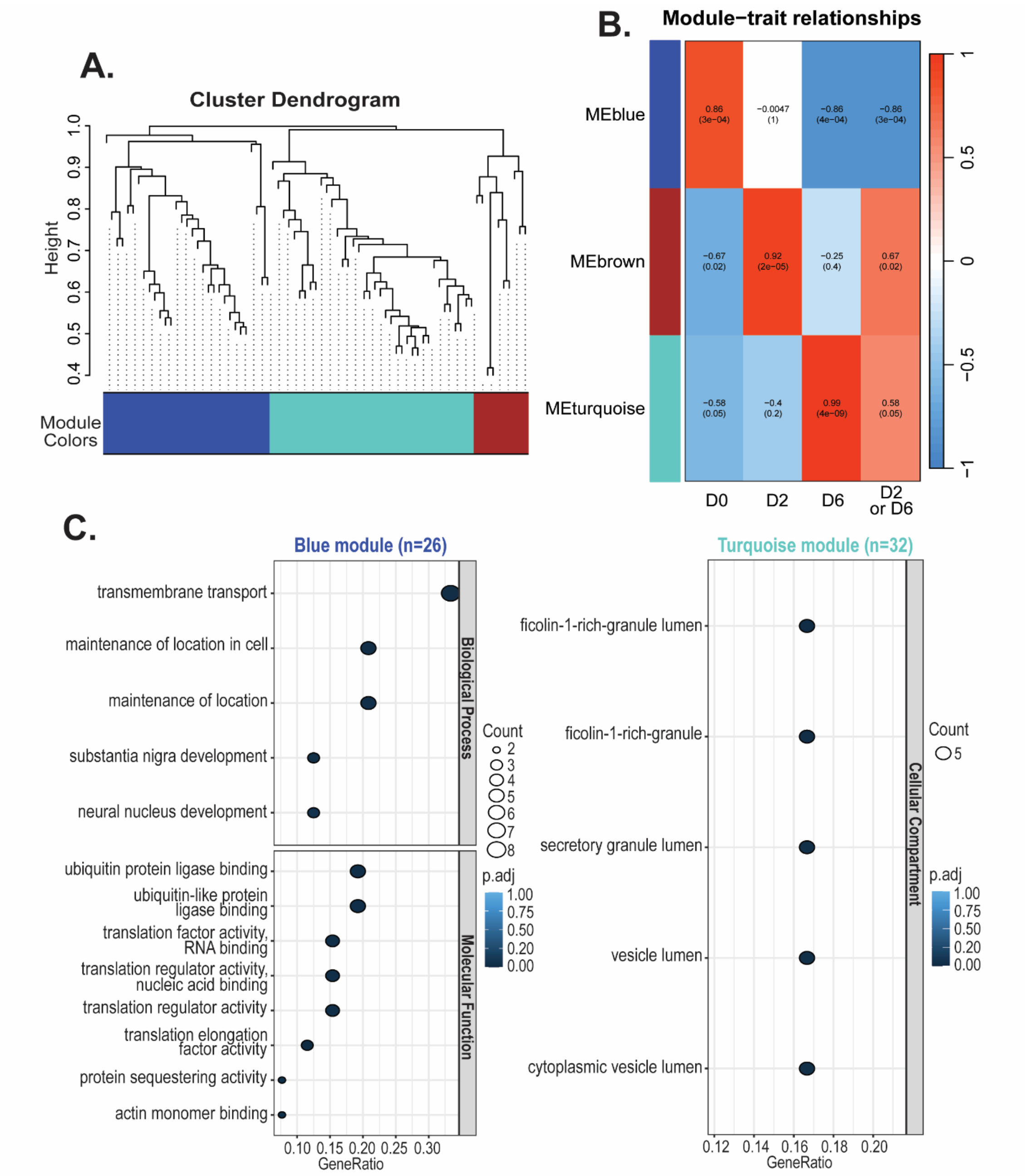

Figure S13: Distinct Kme peptide co-expression modules and associated functions revealed through WGCNA and GO terms analyses. **(A)** WGCNA dendrogram and **(B)** module-trait relationship correlation of Kme peptide co-expression modules identified by WGCNA analysis of differentially abundant Kme peptides. **(C)** Enriched GO terms for proteins corresponding to differentially abundant Kme peptides associated with the following modules: blue (enriched expression differentiation day 0) and turquoise (enriched expression differentiation day 6). There were no enriched biological processes, cellular compartments, or molecular functions for the brown module (enriched expression differentiation day 2).

bioRxiv preprint doi: https://doi.org/10.64898/2025.12.31.696910; this version posted January 2, 2026. The copyright holder for this preprint (which was not certified by peer review) is the author/funder, who has granted bioRxiv a license to display the preprint in perpetuity. It is made available under aCC-BY 4.0 International license.

	Trypsin/Lys-C			Chymotrypsin			Trypsin/Lys-C & Chymotrypsin Combined		
	# Kme peptides	# Kme sites	# Corr. proteins	# Kme peptides	# Kme sites	# Corr. proteins	# Kme peptides	# Kme sites	# Corr. proteins
**Detected**	212	191	155	91	88	73	303	273	222
**Quantified**	120	108	84	26	24	18	146	127	98
**Differentially Abundant**	75	70	54	4	4	3	79	74	57

Table S1

bioRxiv preprint doi: https://doi.org/10.64898/2025.12.31.696910; this version posted January 2, 2026. The copyright holder for this preprint (which was not certified by peer review) is the author/funder, who has granted bioRxiv a license to display the preprint in perpetuity. It is made available under aCC-BY 4.0 International license.

Kme Site	Protein	Protein Description	Kme site reported in PhosphoSite?	Location of methylated K within protein	Reported impact of Kme event
CaM3-K116-m3	Calmodulin-3	Calcium effector protein	Yes	EF-hand calcium binding domain (IPR002048)	CAMKMT-mediated tri-methylation of calmodulin at K116 has been demonstrated to attenuate its activation of calmodulin-dependent NAD kinase *in vitro* (PMID:3003072).
TUBB2A-K19-m3	Tubulin beta-2A chain	Cytoskeletal protein (major constituent of microtubules)	Yes	GTP-binding domain conserved among tubulin proteins (PMID:33800665)	N/A
CFL1-K144-m1	Cofilin-1	F-actin depolymerization factor	No	Cofilin, Destrin, and related actin depolymerizing factors (ADF) domain (IPR017904)	N/A
NOVA2-K424-m3	RNA-binding protein Nova-2	RNA binding alternative splicing regulator	No	KH homology RNA-binding domain (IPR004087)	N/A
NOVA2-K427-m3			No		N/A
CRNKL1-K445-m1	Crooked neck-like protein 1	Pre-mRNA splicing factor	No	Half-a-Tetratricopeptide (HAT) repeat (IPR055430); Mediates interaction with HSP90 (Uniprot manual assertion)	N/A
CUX1-K1266-m2	Homeobox protein cut-like 1	Transcription factor	No	Homeodomain (IPR001356)	N/A
Signaling Cytoskeletal RNA splicing factor Transcription factor					

Table S2

Kme Site	Gene SFARI Score	Pathogenicity Designation	Variant Type	ClinVar Accession	Kme site in PhosphoSite ?	Location of methylated K within protein	Reported impact of Kme event
HNRNPU-K462-m3	1S	-Uncertain significance for Developmental and epileptic encephalopathy, 54	Missense variant (p.Lys462Arg)	VCV001943455.4	No	B30.2/SPRY domain (IPR001870)	N/A
ETFB-K200-m3	2	-1 report Uncertain significance and 1 report Likely pathogenic for Multiple acyl-CoA dehydrogenase deficiency	Splice acceptor variant (HGVS coding: c.598-1del)	VCV000808634.30	Yes	Electron transfer flavoprotein (ETF) beta domain (IPR033948)	METTL20-mediated methylation of ETFB at K200 and K203 inhibits ETFB-mediated electron transfer from acyl-CoA dehydrogenases (PMID: 25416781).
		-Uncertain significance for Multiple acyl-CoA dehydrogenase deficiency	Missense variant (p.Lys200Glu)	VCV000459960.8			
EEF1A2-K55-m2/3	S	-Uncertain significance for Neurodevelopmental disorder	Missense variant (p.Lys55Arg)	VCV001701882.1	Yes	Translational (tr)-type GTP-binding domain (IPR000795)	METTL13-mediated eEF1A methylation at K55 increases eEF1A GTPase activity *in vitro* and stimulates protein synthesis in cancer cells (PMID: 30612740).

Table S3
